# An assessment of the factors contributing to the unavailability of drugs at outpatient pharmacy of tertiary care hospital: an observational study

**DOI:** 10.12688/f1000research.139510.1

**Published:** 2023-10-09

**Authors:** Abhishek Bakare, Aditya Bhargav

**Affiliations:** 1 Department of Hospital Administration., Datta Meghe Institute of Higher Education and research, Sawangi Meghe, Wardha., Maharashtra, 442001, India

**Keywords:** Outpatient pharmacy, drug, tertiary care hospital, unavailability of drug, opd, invalid prescription

## Abstract

Introduction

Throughout history and up until the present, there has been a medicine shortage. In the early 1920s, there was a shortage of insulin, which is when drug scarcity first appeared in the records. Drug shortages are now more prevalent globally than they were back then.

The goal of this essay is to pinpoint the key components that make up a definition for medication shortages and to pinpoint the circumstances that should be considered when reporting drug shortages in databases. Understanding the factors that led certain organizations to create their own definition of a medicine shortage was crucial for achieving these goals.

The pharmaceutical regulatory environment can be connected to several reasons why there are medication shortages, including parallel trading, quality standards, and business choices to halt or reduce manufacturing. The many rules governing medicine shortages have not yet been the subject of a thorough investigation. This protocol’s objective is to analyze the pertinent legislative and regulatory frameworks in the European pharmaceutical system that affect medication shortages. The objectives of the study will be the non–availability of drugs at an outpatient pharmacy and to analyze the reason of non–availability of drugs.

Methods

An observational study will be adopted in this study. It includes a collection of data from the patient coming to the outpatient pharmacy of AVBRH Sawangi (Meghe) Wardha.

Expected result

It can lead to delayed treatment for patients seeking alternative medication. It can also lead to increased healthcare costs if patients seek alternative treatments that are more expensive or require additional medical care. The unavailability of drugs can also lead to frustration and anxiety for patients who need medication to manage their health condition. It can also negatively impact the reputation of the hospital.

## Introduction

Throughout history and up until the present, there has been a medicine shortage. In the early 1920s, there was a shortage of insulin, which is when drug scarcity first appeared in the records. Drug shortages are now more prevalent globally than they were back then. ^1^

The goal of this protocol is to pinpoint the key components that make up a definition for medication shortages and to pinpoint the circumstances that should be considered when reporting drug shortages in databases. Understanding the factors that led certain organizations to create their own definition of a medicine shortage was crucial for achieving these goals. ^2^

The pharmaceutical regulatory environment can be connected to several reasons why there are medication shortages, including parallel trading, quality standards, and business choices to halt or reduce manufacturing. The many rules governing medicine shortages have not yet been the subject of a thorough investigation. This protocol’s objective is to analyze the pertinent legislative and regulatory frameworks in the European pharmaceutical system that affect medication shortages. ^3^

Local ordering problems, regional or national distribution problems, or manufacturing problems can all lead to supply problems that could lead to shortages on a regional or national scale. The United States has a complicated just-in-time inventory system that is used to distribute medications. A cost-saving method used to reduce the costs of carrying extra inventory is just-in-time inventory. This demonstrates that there is generally no product overstock anywhere throughout the supply chain. Generally, wholesalers are used to distribute the pharmaceuticals that manufacturers make. AmerisourceBergen, Cardinal, and McKesson are the top three wholesalers in the US. These wholesalers' distribution centers are dispersed throughout the nation. ^4^

Nonetheless, it would be mistaken to ignore the reality that medicine shortages are also moral and political challenges if we just saw them as technical and economic events. Two things are intended by this: First, medicine shortages affect governments' and practitioners' ability to uphold their moral commitments to citizens and society when they occur Namely, to offer benefits, reduce damage, and encourage equity. Second, Social values lead to pharmaceutical shortages, particularly the choices we've made regarding what we value most in the pharmaceutical and biotechnology sectors, in government regulations, and in health care. These insights are significant because they emphasize the moral and political need to forcefully combat medicine shortages and the requirement that those who do so do it in politically and morally astute ways. ^5^

The key factor causing medicine shortages in Saudi Arabia is the absence of a mechanism for early warning that may send out notifications about impending shortages. There is no penalty for failure to alert the Saudi Food and Drug Administration (SFDA) of any anticipated at a minimum six months prior to any shortages as there are currently no rules requiring such notification from pharmaceutical businesses and importers. Likewise, there are no effective sanctions against authorized importers and distributors of pharmaceutical firms who disregard Saudi government restrictions. Additional causes that cause medicine shortages in Saudi Arabia include inadequate local pharmaceutical production, flawed supply chain management systems, poor profit margins for some essential critical pharmaceuticals, tight regulatory requirements for biological medical goods, and excessive reliance on medication imports. ^6^

### Ethics and consent

The synopsis for this study has been sent to the IEC (DMIHER (DU)/IEC/2023/1162) in the institute for ethical approval and is currently under consideration. Written consent will be taken from the participants who are participating in the study.

### Study design

This study will be an observational study. The study will be conducted in Acharya Vinoba Bhave Rural Hospital. The duration of the study will be two years.

### Sample size

The sample size of the study is 354. The data of this study is going to be conducted from an outpatient pharmacy and self-questionnaires. Questions will be given to the participant for data analysis.

### Sample size formula

$$N\geq \frac{Z_{1-\alpha /2}^{2}\times p\;\left(1-p\right)}{d^{2}}$$

Where,

Z ^2^_1_ – α/2 is the level of significance at 5% i.e. 95%

Confidence interval = 1.96

Alpha (α) = 0.05

Estimate proportion (p) = 0.36 (48 out of 60 were the patient satisfying result as per reference article)

Estimate error (d) = 0.05

$$N=\frac{{\left(1.96\right)}^{2}*\left(0.36\right)*\left(1-0.36\right)}{{\left(0.05\right)}^{2}}$$

N = 354

N = 354 subjects needed in the study

### Inclusion and exclusion criteria

Included in this study will be the Patient willing to give a consent form, Patient having a valid prescription. And the Patient is willing to comply with the study protocol. excluded from the study will be Inpatient Invalid prescriptions.

Survey and questionary development of a verified self-questionary and direct open-ended questionary for outpatients who will be coming to the outpatient pharmacy.

### Data collection techniques

The primary data for this study will be collected through an observational approach. This will involve in-depth interviews with patients and pharmacy staff, as well as observations of the pharmacy and its operations. Data will also be collected through hospital records to track the availability of drugs over time.

### Data analysis

Data will be analyzed by using simple descriptive statistics. Data will be presented in graphical, tables, charts, etc. Microsoft Excel will be used for data analysis.

### Expected result

It can lead to delayed treatment for patients seeking alternative medication. It can also lead to increased healthcare costs if patients seek alternative treatments that are more expensive or require additional medical care. The unavailability of drugs can also lead to frustration and anxiety for patients who need medication to manage their health conditions. It can also negatively impact the reputation of the hospital.

## Discussion

An observational study carried out in Belgium, on 30 October 2015 found that currently, there are more frequent medicine shortages. Although numerous definitions of “drug shortages” are provided in legislation, by various organizations, authorities, and other initiatives, a thorough analysis of the issue is still required. To properly interpret national databases and the findings of scientific investigations and allow for international comparison, it is crucial to understand the fundamental definition of drug shortages. The goal is to establish the many components that should be considered in a standardized definition for drug shortages in the European Union (EU) and to identify the various circumstances in which drug shortages should be reported. The methods employed involved searching scientific databases and grey literature for definitions of drug shortages. Similar subjects were found, and organizations were approached to develop the definitions' supporting arguments. According to the findings, there are over 20 different definitions of medicine shortages. There is a discrepancy between the terminology used for reporting drug shortages and the definitions used in general. The definitions' elements—such as when a supply issue turns into a scarcity of drugs, whether they are permanent or temporary, their typology, and their duration—show both parallels and differences. There are four levels at which a supply issue becomes a shortage: (i) demand side; (ii) supply side; (iii) drug delivery; and (iv) drug availability. Definitions of drug shortages do not usually include permanent drug discontinuations. Some definitions solely consider medications needed to treat serious illnesses or medications for which there is no effective substitute. The observed time intervals ranged from one day to twenty days. The study's conclusion was to enable worldwide benchmarking, a standardized definition of drug shortages must be established, together with a list of the situations in which reporting drug shortages is preferred. A number of studies related to shortages in outpatient pharmacy were reported. ^7^^-^^11^ This essay can be used as a reference to highlight all the various factors that should be considered while formulating a definition that will be used throughout the EU. ^3^

### Dissemination

The study will be published in an institutional-indexed journal.

### Study status

The study is under process with 50% Completion.

## Data Availability

No data associated with this article.
