# Supplementary material for: An assessment of the factors contributing to the unavailability of drugs at outpatient pharmacy of tertiary care hospital: an observational study
Source: F1000Res. 2024 Oct 14;12:1287. Originally published 2023 Oct 9. [Version 2] doi: 10.12688/f1000research.139510.2 (PMC11615433; doi:10.12688/f1000research.139510.2)
Supplement: Peer review history

# Reviewer report: Reviewer response for version 2

**DOI:** 10.5256/f1000research.173200.r331620

**Published:** 2024-12-03

**Authors:** Unyime Israel Eshiet

**Affiliations:** 1 University of Uyo, Uyo, Nigeria

Satisfactory.

 Please accept for indexing.

Is the study design appropriate for the research question?

Partly

Is the rationale for, and objectives of, the study clearly described?

Partly

Are sufficient details of the methods provided to allow replication by others?

No

Are the datasets clearly presented in a useable and accessible format?

Not applicable

Reviewer Expertise:

NA

I confirm that I have read this submission and believe that I have an appropriate level of expertise to confirm that it is of an acceptable scientific standard.

# Reviewer report: Reviewer response for version 1

**DOI:** 10.5256/f1000research.152787.r294888

**Published:** 2024-09-09

**Authors:** Unyime Israel Eshiet

**Affiliations:** 1 University of Uyo, Uyo, Nigeria

This is an interesting study and well conceived. The authors have however not explicitly presented the specific objectives of the study. The proposed study designed is not clearly described to ascertain suitability. Also the method is not sufficiently described. Authors should kindly provide details of the data collection instrument(s).

 What are the possible limitations of the study?

Is the study design appropriate for the research question?

Partly

Is the rationale for, and objectives of, the study clearly described?

Partly

Are sufficient details of the methods provided to allow replication by others?

No

Are the datasets clearly presented in a useable and accessible format?

Not applicable

Reviewer Expertise:

NA

I confirm that I have read this submission and believe that I have an appropriate level of expertise to confirm that it is of an acceptable scientific standard, however I have significant reservations, as outlined above.

# Response

**Published:** 2024-10-08

**Authors:** Abhishek Bakare

**Affiliations:** Datta Meghe Institute Of Higher Education And Research, India

Thanks for your valuable time and suggestions. 1. We have added the objectives as suggested. 2. The methodology part has been updated and details about data collection instruments have been added. Also, the limitations of the study are added. Thanks for your kind perusal.

# Reviewer report: Reviewer response for version 1

**DOI:** 10.5256/f1000research.152787.r281983

**Published:** 2024-06-24

**Authors:** Trudy Huyghebaert

**Affiliations:** 1 School of Health and Wellness, Bow Valley College, Calgary, Alberta, Canada

This is a very important topic and I think one of the key aspects of this is with regard to the moral and ethical obligation of drug companies to provide a consistent supply to their medication for patients, which may involve re-evaluating their just-in time approach to inventory as described.

 Overall, this is a very well written protocol. It is a study protocol and there are no results to evaluate  Comments are added below for a few minor revisions: 

 1. Provide an expended definition of parallel trading

 2. The authors refer extensively to the United States system and wholesalers and then segway into Saudia Arabia system.  If possible, please explain more about the correlation (or similarities) between the two systems.

Is the study design appropriate for the research question?

Yes

Is the rationale for, and objectives of, the study clearly described?

Yes

Are sufficient details of the methods provided to allow replication by others?

Yes

Are the datasets clearly presented in a useable and accessible format?

Not applicable

Reviewer Expertise:

quality improvement, primary care, evidence based medicine

I confirm that I have read this submission and believe that I have an appropriate level of expertise to confirm that it is of an acceptable scientific standard.
